# Physiologically relevant orthogonal assays for the discovery of small-molecule modulators of WIP1 phosphatase in high-throughput screens

**DOI:** 10.1074/jbc.RA119.010201

**Published:** 2019-09-03

**Authors:** Victor Clausse, Dingyin Tao, Subrata Debnath, Yuhong Fang, Harichandra D. Tagad, Yuhong Wang, Hongmao Sun, Christopher A. LeClair, Sharlyn J. Mazur, Kelly Lane, Zhen-Dan Shi, Olga Vasalatiy, Rebecca Eells, Lynn K. Baker, Mark J. Henderson, Martin R. Webb, Min Shen, Matthew D. Hall, Ettore Appella, Daniel H. Appella, Nathan P. Coussens

**Affiliations:** ‡ Synthetic Bioactive Molecules Section, Laboratory of Bioorganic Chemistry, NIDDK, National Institutes of Health, Bethesda, Maryland 20892; § National Center for Advancing Translational Sciences, National Institutes of Health, Rockville, Maryland 20850; ¶ Laboratory of Cell Biology, Center for Cancer Research, NCI, National Institutes of Health, Bethesda, Maryland 20892; ‖ Imaging Probe Development Center, NHLBI, National Institutes of Health, Rockville, Maryland 20850; ** Reaction Biology Corporation, 1 Great Valley Parkway, Suite 2, Malvern, Pennsylvania 19355; ‡‡ Francis Crick Institute, 1 Midland Road, London NW1 AT, United Kingdom

**Keywords:** high-throughput screening (HTS), phosphatase, cancer, fluorescence, mass spectrometry (MS), cancer therapy, enzyme kinetics, phosphorylation, oncogene, assay, kinetics, RapidFire, therapeutics, Wip1, fluorescence

## Abstract

WT P53-Induced Phosphatase 1 (WIP1) is a member of the magnesium-dependent serine/threonine protein phosphatase (PPM) family and is induced by P53 in response to DNA damage. In several human cancers, the WIP1 protein is overexpressed, which is generally associated with a worse prognosis. Although WIP1 is an attractive therapeutic target, no potent, selective, and bioactive small-molecule modulator with favorable pharmacokinetics has been reported. Phosphatase enzymes are among the most challenging targets for small molecules because of the difficulty of achieving both modulator selectivity and bioavailability. Another major obstacle has been the availability of robust and physiologically relevant phosphatase assays that are suitable for high-throughput screening. Here, we describe orthogonal biochemical WIP1 activity assays that utilize phosphopeptides from native WIP1 substrates. We optimized an MS assay to quantify the enzymatically dephosphorylated peptide reaction product in a 384-well format. Additionally, a red-shifted fluorescence assay was optimized in a 1,536-well format to enable real-time WIP1 activity measurements through the detection of the orthogonal reaction product, P_i_. We validated these two optimized assays by quantitative high-throughput screening against the National Center for Advancing Translational Sciences (NCATS) Pharmaceutical Collection and used secondary assays to confirm and evaluate inhibitors identified in the primary screen. Five inhibitors were further tested with an orthogonal WIP1 activity assay and surface plasmon resonance binding studies. Our results validate the application of miniaturized physiologically relevant and orthogonal WIP1 activity assays to discover small-molecule modulators from high-throughput screens.

## Introduction

Dysregulation of both protein kinase and protein phosphatase activity is common in many human diseases, including cancer. Approximately 20% of the 189 human protein phosphatases have genetic variants associated with human diseases (1). Activation of oncogenic protein phosphatases or the functional loss of tumor suppressor protein phosphatases can contribute to cancer and these enzymes have been implicated as potential therapeutic targets (2, 3). However, targeting the active sites of protein phosphatases with small molecules has proven challenging, with toxicity, specificity, and pharmacokinetics identified as major obstacles (4–6). Moreover, many enzymes reside in nonactive conformations that may be activated by post-translational modifications; therefore, developing assays to uncover small molecules that stabilize active conformations is also of a great therapeutic interest (7).

WT P53-Induced Phosphatase 1 (WIP1),^5^ encoded by the *PPM1D* gene, is a serine/threonine protein phosphatase belonging to the PPM (formerly PP2C) family. WIP1 was first described as a protein induced by P53 in response to ionizing radiation (8). Like other PPM family members, the phosphatase activity of WIP1 is Mg^2+^/Mn^2+^ dependent and is insensitive to okadaic acid. WIP1 dephosphorylates several proteins involved in the DNA damage response pathway. Following P53 activation by ATM or ATR, the levels of WIP1 are increased and it acts on many targets, including P53 (9), ATM (10), CHK1 (9), CHK2 (11), P38 (12), and H2AX (13–16). WIP1 has been described as an oncogene (17–19) and its amplification has been reported in several human cancers, including breast (20), ovarian clear cell carcinoma (21), glioma (22), neuroblastoma (23), and medulloblastoma (79). However, overexpression of WIP1 has been shown to sensitize P53-negative cells to chemotherapy and to protect normal tissues during the treatment, suggesting that WIP1 can have tumor suppressor properties (24, 25). The role of WIP1 in DNA damage response and its action as an oncogene or tumor suppressor, depending on the P53 status of cancer cells, implicate WIP1 as a potential therapeutic target (26). Our group has shown that substrate-based thioether cyclic peptide inhibitors can be developed with low micromolar potency (27, 28); however, these inhibitors suffer from a poor selectivity within the PPM family. GSK2830371, a potent allosteric inhibitor of WIP1, was demonstrated to have good potency and selectivity (29); however, it does not show favorable pharmacokinetics (30). To our knowledge, no small-molecule activator of WIP1 has yet been described.

**Figure 1.**
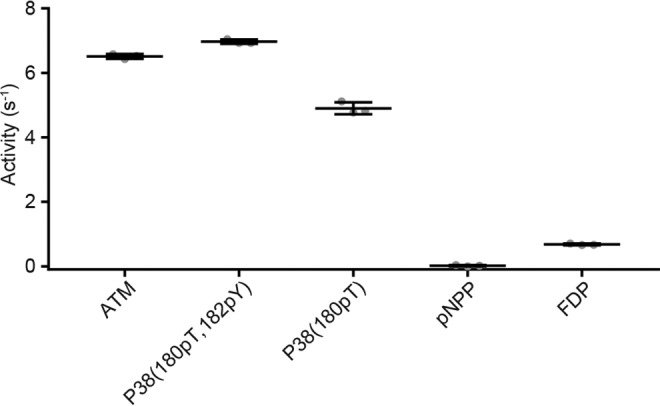
**WIP1 is more active against phosphopeptide substrates compared with small molecules commonly used as substrates to measure phosphatase activity.** Data show the mean ± S.D. for triplicate activity measurements using a BIOMOL Green assay, where all substrates were tested at 100 μm. The activity of WIP1 toward p-nitrophenyl phosphate (pNPP) is several hundred-fold lower compared with phosphopeptides derived from WIP1 substrates ATM and P38. Similarly, the activity of WIP1 toward fluorescein diphosphate (FDP) is 7–10-fold lower compared with the ATM and P38 phosphopeptide substrates.

A major challenge in developing small-molecule modulators for phosphatases is the paucity of assays suitable for high-throughput screening (HTS) that utilize physiologically relevant substrates. Early *in vitro* phosphatase assays used malachite green for a colorimetric readout (31–33) and have been successfully adapted for use in HTS (34, 35). More commonly, HTS assays utilize artificial nonpeptide small-molecule phosphatase substrates, such as the chromogenic substrate *p*-nitrophenyl phosphate (pNPP) (35–37) or the fluorogenic substrates: 4-methylumbelliferyl phosphate (MUP), 3-O-methylfluorescein phosphate (OMFP) (38), 6,8-difluoro-4-methylumbelliferyl phosphate (DiFMUP) (37, 39), and 3,6-fluorescein diphosphate (FDP) (40). For example, a biochemical assay measuring the hydrolysis of FDP by WIP1 contributed to the discovery of GSK2830371 (29). However, compared with phosphopeptides from native substrates, the activity of WIP1 toward the small-molecule substrates, pNPP and FDP, is substantially lower (Fig. 1). Additionally, the use of artificial substrates in HTS can lead to the identification of hits with reduced biological and pharmacological relevance. For example, discrepancies in the potencies of Nucleotide Pyrophosphatase/Phosphodiesterase 1 (NPP1) inhibitors, depending on the substrate used, have been reported (41, 42). Full-length phosphoprotein substrates need to be co-expressed with a kinase or phosphorylated *in vitro* and often show poor solubility, which make them challenging for HTS applications (43, 44). Phosphopeptide substrates have a greater physiological relevance than artificial small-molecule substrates and have also been successfully incorporated into WIP1 activity assays for HTS. One study utilized the IQ^TM^ Phosphatase Assay technology (Pierce), based on fluorescence intensity quenching of a fluorophore-labeled peptide after a proprietary iron-containing compound binds the phosphoryl group (45). Another study applied AlphaScreen technology (PerkinElmer) to measure phosphorylation of a biotinylated phospho-P38 peptide with a mouse phospho-specific anti-P38 mAb (46). The AlphaScreen signal is proportional to the proximity of streptavidin-coated donor beads and anti-mouse IgG-coated acceptor beads. Both of these assay formats required conjugation of a label to the phosphopeptide substrate (rhodamine fluorophore or biotin), which can alter the interaction between the substrate and enzyme. Also, both assays measure the substrate concentration (phosphorylated peptide) rather than the reaction products (dephosphorylated peptide or P_i_), which means that the assay sensitivity is limited in the early phase of the reaction with 15–20% substrate turnover. Here we report the development, optimization, and validation of orthogonal WIP1 activity assays using unmodified native phosphopeptide substrates. The first assay uses RapidFire MS to quantify the dephosphorylated reaction product in a 384-well format. The second assay applies a previously described phosphate binding protein with a red-shifted fluorescence reporter (47, 48) to enable real-time measurements of the orthogonal reaction product, P_i_. Both optimized assays were validated by screening the NCATS Pharmaceutical Collection (NPC) (49) with a quantitative high-throughput screening (qHTS) format (50). Confirmed hits from the primary screens were further evaluated by a variety of additional assays to characterize their activities, including binding studies by surface plasmon resonance (SPR).

## Results

### Development and optimization of a RapidFire MS assay for WIP1 activity

**Figure 2.**
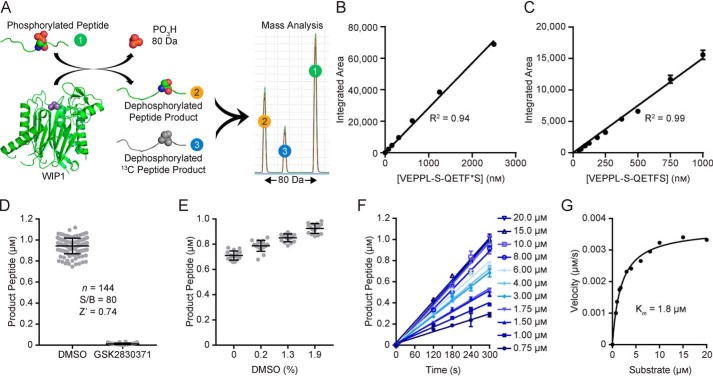
**Optimization of a RapidFire mass spectrometry (MS) assay.** *(A)*, RapidFire MS assay scheme for quantification of the dephosphorylated peptide product from the WIP1 reaction. *(B)*, The integrated areas from MS analysis of the heavy-labeled product peptide **4** are linearly related to peptide concentrations between 0 and 2.5 μm (R^2^ = 0.94), which serves as a calibration standard for the WIP1 reaction product. *(C)*, A range of WIP1 reaction product peptide concentrations were analyzed and the limit of quantitation was determined to be 28.3 nm. *(D)*, The optimized assay is robust with an 80-fold signal-to-background value between the vehicle (DMSO) and 100% inhibition (GSK2830371) controls and a Z′-factor value of 0.74. Individual data points are shown and bars indicate mean ± S.D. (*n* = 144). *(E)*, Titrations of DMSO demonstrated that assay performance is not diminished by increased vehicle concentrations up to 1.9% (mean ± S.D.; *n* = 16). *(F)*, Initial velocities for WIP1 with a range of P53 phosphopeptide substrate **3** concentrations as determined by Rapidfire MS. *(G)*, Velocities measured from the data in *(F)* were plotted and fit to the Michaelis-Menten equation to estimate the *K_m_* at 1.8 μm.

We developed a sensitive assay in a 384-well format to measure WIP1 activity toward a phosphopeptide substrate using RapidFire MS to quantify the dephosphorylated reaction product (Fig. 2*A*). Following quenching of the reaction with formic acid, accurate product quantification was enabled by spiking assay samples with 1 μm of a ^13^C-labeled product analyte as an internal calibration standard. The integrated detection peak areas were linear for concentrations of the peptide standard between 0 and 2.5 μm (R^2^ = 0.94, Fig. 2*B*). The limit of quantitation (LOQ) for the reaction product peptide was determined to be 28.3 nm (Fig. 2*C*). The previously described WIP1 inhibitor GSK2830371 was used as a control for 100% enzyme inhibition and the optimized assay was robust with a signal-to-background value of 80 and Z′-factor value of 0.74 (Fig. 2*D*). Addition of DMSO vehicle up to 1.9% (v/v) did not reduce assay performance (Fig. 2*E*). The optimized RapidFire MS WIP1 activity assay was used to evaluate the initial reaction velocities with substrate concentrations ranging from 0.75 to 20 μm (Fig. 2*F*). The data were fit to the Michaelis-Menten model with an apparent *K_m_* value of 1.85 ± 0.14 μm (Fig. 2*G*). The optimized instrument settings for RapidFire MS are shown in Table 1.

**Table 1:** **RapidFire MS settings and information about precursor and product ions for each multiple reaction monitoring transition**

	VEPPLpSQETFS	VEPPLSQETF*S*^a^*	VEPPLSQETFS
Peptide number	3	4	5
Precursor ion	657.3	620.5	617.3
MS1 resolution	Unit	Unit	Unit
Product ion	1061.5, 253.2	611.6, 536.3	536.4, 253.2
MS2 resolution	Wide	Wide	Wide
Dwell (ms)	10	10	10
Fragmentor (V)	135	135	135
Collision energy (V)	12, 16	8, 10	10, 14
Cell acceleration (V)	5	5	5
Polarity	Positive	Positive	Positive

*^a^* F*, ring ^13^C_6_-labeled phenylalanine.

### Development and optimization of a red-shifted, real-time fluorescence assay for WIP1 activity

**Figure 3.**
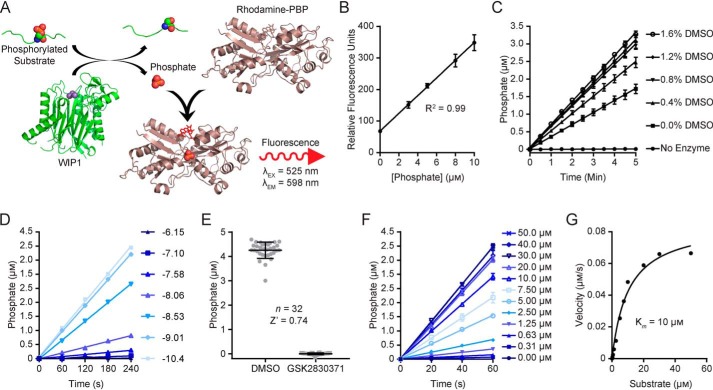
**Optimization of a red-shifted fluorescence-based WIP1 kinetic activity assay with rhodamine-labeled phosphate binding protein (Rh-PBP).** (*A*), The Rh-PBP assay scheme for quantification of the WIP1 reaction product, P_i_. (*B*), The Rh-PBP (10 μm) provides a linear fluorescence readout of P_i_ concentrations (mean ± S.D.; *n* = 6). (*C*), Titrations of DMSO demonstrated that the assay performance is not diminished by vehicle concentrations up to 1.6% (mean ± S.D.; *n* = 6). Concentrations of the phosphate reaction product were determined from the standard curve (*B*), which was included in the same microwell plate. (*D*), WIP1 reaction rates are reduced with increasing concentrations of the inhibitor GSK2830371 (concentrations are indicated as Log molar values for *n* = 1). (*E*), The optimized assay is robust with a Z′-factor value of 0.74 for vehicle (DMSO) and 100% inhibition (GSK2830371) controls. (*F*), Initial velocities for WIP1 with a range of P53 phosphopeptide substrate **3** concentrations as determined with the Rh-PBP assay. (*G*), Velocities measured from the data in (*F*) were plotted and fit to the Michaelis-Menten equation to estimate the apparent *K_m_* value of 10 μm.

Using the previously described rhodamine-labeled phosphate binding protein (Rh-PBP) (47, 48), we developed a novel red-shifted fluorescence assay as an orthogonal method for identifying small-molecule modulators of WIP1. This assay technology enables the use of any phosphopeptide substrate and provides a real-time measurement of WIP1 activity through detection of the reaction product, P_i_ (Fig. 3*A*). Moreover, the high sensitivity and dynamic range enabled miniaturization to a 1,536-well format. With 10 μm Rh-PBP, the red-fluorescence readout was linear for phosphate concentrations between 0 and 10 μm (Fig. 3*B*), with a 5-fold dynamic range. Inclusion of a phosphate standard curve in each microwell plate enables quantification of the phosphate concentration from the measured fluorescence. We measured the activity of WIP1 toward a phosphopeptide substrate derived from P53 as the rate of product formation over time and observed no reduction in the activity in the presence of DMSO vehicle concentrations up to 1.6% (v/v) (Fig. 3*C*). As expected, GSK2830371 reduced the WIP1 reaction rate in a dose-dependent manner (Fig. 3*D*). GSK2830371 was used as a control for 100% enzyme inhibition and the optimized assay was robust with a Z′-factor value of 0.74 (Fig. 3*E*). Quantification of real-time activity is convenient for initial velocity measurements (Fig. 3*F*) to evaluate Michaelis-Menten kinetics (Fig. 3*G*), which enabled an estimation of the apparent *K_m_* of the phosphopeptide substrate at 10 μm.

### Validation of the optimized orthogonal WIP1 activity assays by pilot-scale qHTS of the NCATS Pharmaceutical Collection

**Figure 4.**
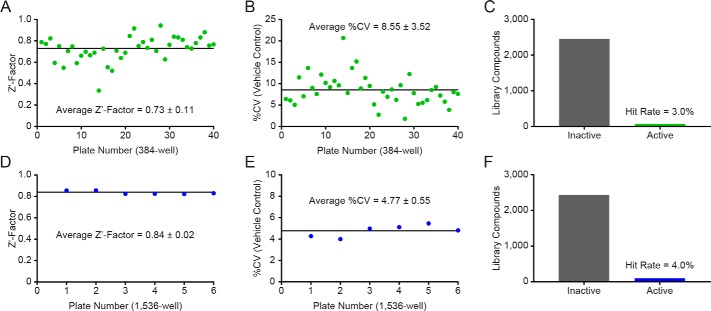
**Quantitative high-throughput screening with orthogonal WIP1 primary assays against the NCATS Pharmaceutical Collection.** (*A–C*), Statistics from the qHTS primary screen with the RapidFire MS assay at 5 concentrations. (*A*), The assay performance of the screen was robust with an average Z′-factor value of 0.73 among forty 384-well plates. (*B*), The average %CV for the DMSO control was 8.55. (*C*), Among the 2,535 compounds tested, 76 inhibited WIP1 beyond 50% (3.0% hit rate). (*D–F*), Statistics from the qHTS primary screen with the Rh-PBP assay at 3 concentrations. (*D*), The assay performance of the screen was robust with an average Z′-factor value of 0.84 among six 1,536-well plates. (*E*), The average %CV for the DMSO control was 4.77. (*F*), Among the 2,535 compounds tested, 102 inhibited WIP1 beyond 50% (4.0% hit rate).

The optimized RapidFire MS WIP1 activity assay was validated by screening the NPC (49) using qHTS (50) at 5 concentrations in 384-well plates (Table 2). The overall quality of the primary screen across 40 plates was high, with an average Z′-factor value of 0.73 ± 0.11 (Fig. 4*A*) and an average coefficient of variation (CV) for the DMSO control of 8.55 ± 3.52% (Fig. 4*B*). Of the 2,535 compounds evaluated in the primary screen, 76 compounds were active with a maximum response ≥50%, corresponding to a hit rate of 3.0% (Fig. 4*C*).

**Table 2:**
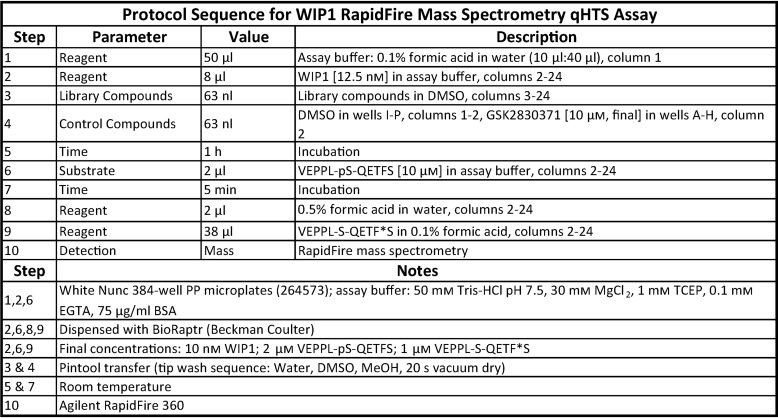
**Optimized protocol for the WIP1 RapidFire MS assay used for qHTS**

To validate the optimized Rh-PBP WIP1 activity assay, the NPC was screened using qHTS at 3 concentrations in a 1,536-well format (Table 3). The quality of the primary screen was high across 6 plates, with an average Z′-factor value of 0.84 ± 0.02 (Fig. 4*D*) and an average coefficient of variation (CV) for the DMSO control of 4.77 ± 0.55% (Fig. 4*E*). Among 2,535 compounds, 102 compounds showed activity with a maximum response ≥50%, corresponding to a hit rate of 4.0% (Fig. 4*F*).

**Table 3:**
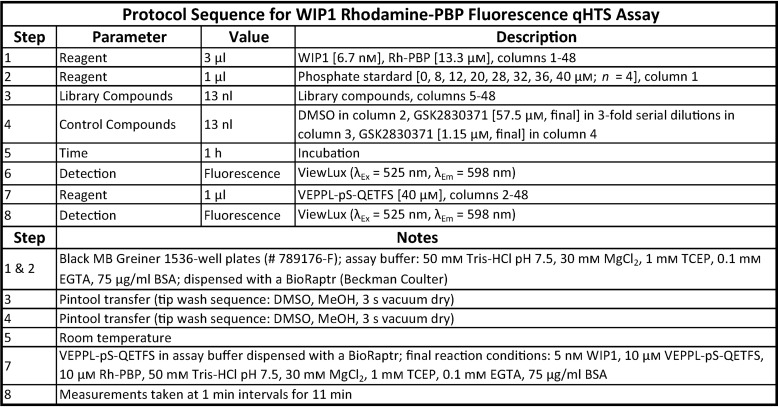
**Optimized protocol for the WIP1 activity assay using the phosphate-binding protein with a red-shifted readout**

### Hit confirmation and secondary screening

**Figure 5.**
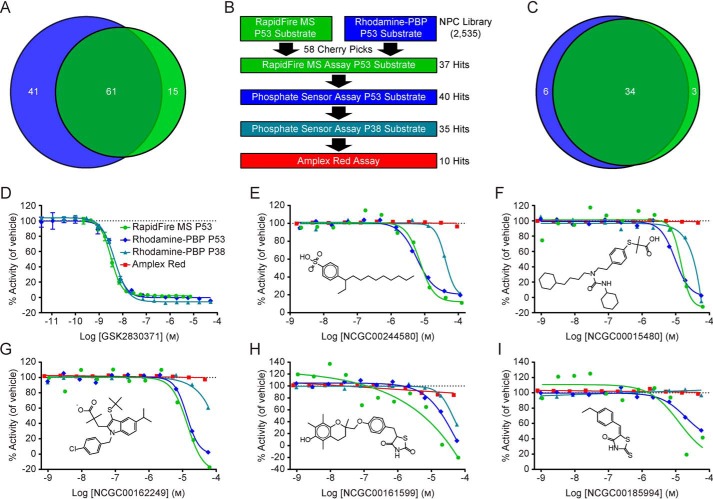
**Primary and secondary screening of the NCATS Pharmaceutical Collection (NPC).** (*A*), Sixty-one common hits were identified by the orthogonal WIP1 activity assays in the qHTS against the NPC library. The Rh-PBP assay (*blue*) had a higher overall hit rate than the RapidFire MS assay (*green*). (*B*), Among the 61 common hits in the primary screens, 58 compounds were selected as cherry picks. The cherry picks were further evaluated in 11-point dose response with the primary assays, the Rh-PBP assay with a P38 peptide substrate **1**, and the Amplex Red assay to identify compounds capable of redox cycling. The number of compounds showing activity (hits) from each assay is indicated on the right. (*C*), Among the 58 cherry picks, 34 compounds were confirmed by both the Rh-PBP assay (69% confirmation rate, blue) and the RapidFire MS assay (64% confirmation rate, green). (*D–I*), Quantitative HTS data from the primary and secondary assays. None of the selected compounds show activity in the Amplex Red assay (red squares). (*D*), GSK2830371 inhibits WIP1 with a low nanomolar potency and was used as a control for the WIP1 activity assays. The data are shown as the mean value ± S.D. for the RapidFire MS assay with the P53 substrate **3** (green circles, IC_50_ = 3.5 nm, Hill slope = −1.9, *n* = 3) and the Rh-PBP assay with the P53 substrate **3** (blue diamonds, IC_50_ = 4.9 nm, Hill slope = −1.7, *n* = 2) or P38 substrate **1** (teal triangles, IC_50_ = 5 nm, Hill slope = −1.5, *n* = 2). (*E*), NCGC00244580 inhibits WIP1 activity toward the P53 substrate **3** with the RapidFire MS assay (IC_50_ = 7.3 μm, Hill slope = −2.1) or Rh-PBP assay (IC_50_ = 5.9 μm, Hill slope = −1.7). The Rh-PBP assay shows that NCGC00244580 also inhibits WIP1 activity against the P38 substrate **1** (IC_50_ = 42 μm, Hill slope = −3). (*F*), NCGC00015480 inhibits WIP1 activity similarly according to the RapidFire MS assay (IC_50_ = 14 μm, Hill slope = −3.1) or the Rh-PBP assay with the P53 substrate **3** (IC_50_ = 9.6 μm, Hill slope = −2) or P38 substrate **1** (ambiguous fit). (*G*), NCGC00162249 inhibits WIP1 activity against the P53 substrate **3** as shown by RapidFire MS (IC_50_ = 14 μm, Hill slope = −1.9) or Rh-PBP (IC_50_ = 13 μm, Hill slope = −2.4) assays, with partial activity at high concentrations with the P38 substrate **1**. (*H*), The activity of WIP1 toward the P53 substrate **3** is inhibited by NCGC00161599 as shown by the RapidFire MS (IC_50_ = 11 μm, Hill slope = −0.28) and Rh-PBP (IC_50_ = 51 μm, Hill slope = −0.1) assays. The activity of WIP1 against the P38 substrate **1** was partially inhibited (IC_50_ = 44 μm, Hill slope = −2). (*I*), Although no inhibition was apparent with the P38 substrate **1**, NCGC00185994 inhibited WIP1 activity against the P53 substrate **3** according to the RapidFire MS (IC_50_ = 13 μm, Hill slope = −1) and Rh-PBP (IC_50_ = 20 μm, Hill slope = −1.2) assays.

Although the hit rates of the two assays were different, 61 compounds were active in both primary qHTS screens (Fig. 5*A*). From this list of common hits, 58 compounds were selected as cherry picks and prepared in 11-point concentration series from library stock solutions for confirmation and further analysis (Fig. 5*B*). The primary assays showed similar confirmation rates, with 64% confirmed by the RapidFire MS assay and 69% confirmed by the Rh-PBP assay. There was excellent agreement among the two assays, which confirmed the activities of 34 compounds, for a combined confirmation rate of 59% (Fig. 5*C*). In addition to the confirmation assays, the cherry picks were evaluated by the Rh-PBP assay with an orthogonal phosphopeptide substrate derived from P38. Thirty-five compounds were identified as active with this secondary assay, which is a hit rate of 60% and is slightly lower than either of the primary assays (Fig. 5*B*). Finally, an amplex red assay was performed in the presence of reducing agents to identify compounds capable of redox cycling (51). Ten compounds demonstrated redox activity beyond a threshold of 3σ (Fig. 5*B*), two of which had been confirmed as active compounds in both primary assays.

The previously described WIP1 inhibitor GSK2830371 (29) was used as a control for the RapidFire MS and Rh-PBP assays. As expected, the inhibitor showed similar dose-dependent inhibition of WIP1 for all three assays (MS with P53 phosphopeptide, Rh-PBP with P53 phosphopeptide and Rh-PBP with P38 phosphopeptide), with an IC_50_ value of ∼5 nm (Fig. 5*D*). Among the 34 confirmed hits, five inhibitors that did not show redox cycling activity in the amplex red assay were selected for additional studies. The most potent inhibitor, NCGC00244580, showed very similar dose-response curves for the RapidFire MS assay (IC_50_ = 7.3 μm, Hill slope = −2.1) and the Rh-PBP assay (IC_50_ = 5.9 μm, Hill slope = −1.7), both of which utilized the P53 substrate (Fig. 5*E*). With the P38 substrate, the Rh-PBP assay showed a similar dose-dependent inhibition, but with a ∼7-fold weaker potency (IC_50_ = 42 μm, Hill slope = −3). Overall, the three WIP1 activity assays displayed comparable sensitivity in the single or double-digit micromolar range (Table 4). Indeed, the inhibitor NCGC00015480 showed an IC_50_ of 11 μm to 31 μm, depending on the substrate peptide used (Fig. 5*F*). The WIP1 inhibitor NCGC00162249 showed complete and comparable dose-dependent inhibition with RapidFire MS (IC_50_ = 14 μm, Hill slope = −1.9) and Rh-PBP (IC_50_ = 13 μm, Hill slope = −2.4) assays using the P53 substrate, but only partial activity at the highest concentrations with the P38 substrate (Fig. 5*G*). Similarly, the potency of NCGC00161599 was in the double-digit micromolar range for the three WIP1 activity assays, with only partial activity from the assay with the P38 substrate (Fig. 5*H*). The fifth compound, NCGC00185994, did not completely inhibit WIP1 at the highest concentrations tested with the P53 substrate and showed no inhibition by the Rh-PBP assay utilizing the P38 substrate (Fig. 5*I*).

**Table 4:**
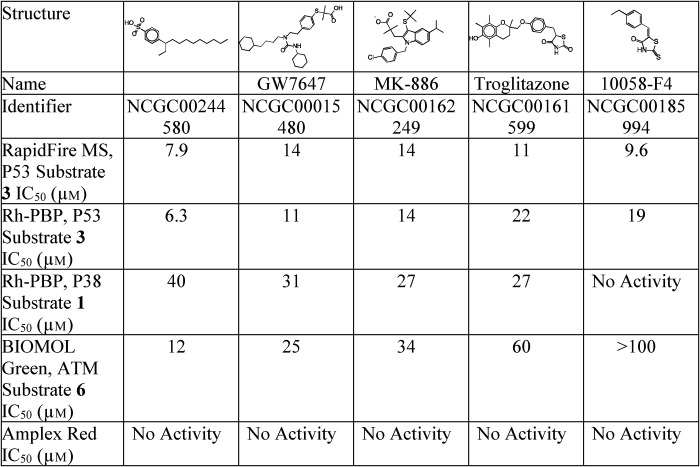
**Biochemical activities of five compounds that inhibit WIP1** Each value is from one experiment with the indicated assay and substrate.

### Further analysis of five inhibitors by an orthogonal WIP1 activity assay

**Figure 6.**
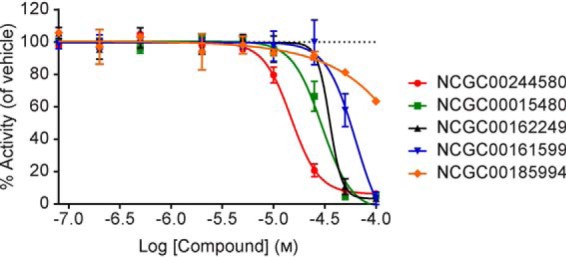
**Analysis of five WIP1 inhibitors by the BIOMOL Green assay with phosphopeptide substrate 6 derived from ATM.** All data are plotted as the mean value ± S.D. for *n* = 2 technical replicates. Beginning with the most potent inhibitor is NCGC00244580 (red circles, IC_50_ = 15 μm, Hill slope = −3.2), NCGC00015480 (green squares, IC_50_ = 30 μm, Hill slope constrained at = −3), NCGC00162249 (black triangles, IC_50_ = 35 μm, Hill slope = −7.1), NCGC00161599 (blue inverted triangles, IC_50_ = 63 μm, Hill slope constrained at = −3), NCGC00185994 (orange diamonds, IC_50_ > 100 μm, Hill slope = −0.9).

To further evaluate the biochemical activities of the five WIP1 inhibitors selected from the primary and secondary screening assays, the BIOMOL Green colorimetric assay was performed with a phosphopeptide substrate derived from ATM. The results of these studies were in general agreement with the screening data and further confirmed the activities of all five compounds (Fig. 6 and Table 4). Similar to the confirmation assays, NCGC00244580 was the most potent compound, whereas the potencies of NCGC00015480, NCGC00162249, and NCGC00161599 were in the double-digit micromolar range. While the previous four compounds fully inhibited WIP1 activity at the highest concentration tested, NCGC00185994 showed only partial inhibition at the highest concentration.

### Binding of inhibitors to WIP1

SPR was used to determine whether the five inhibitors interact directly with WIP1. As expected, the positive control GSK2830371 bound WIP1 with a strong affinity (*K_d_* = 14 nm, Table 5). The SPR data for NCGC00185994 fit reliably to a two-state binding kinetic model, which indicated a dissociation constant of 23 μm (Table 5). The remaining four compounds, NCGC00244580, NCGC00015480, NCGC00162249, and NCGC00161599 bound WIP1 nonspecifically at concentrations near 100 μm (data not shown). It was observed that all inhibitors begin to deviate from stoichiometric binding at concentrations >25 μm, which might be attributed to compound aggregation.

**Table 5:** **Direct binding of NCGC00185994 to WIP1, as measured by surface plasmon resonance, with GSK2830371 as a positive control** All data were fit to a two-state kinetic binding model. Differences in the *R*_max_ values are attributed to the lower immobilization level of channel 1 compared with channel 6.

Compound Name	GSK2830371	NCGC00185994
*k_a_*_1_ (m^−1^ s^−1^)	2.66 × 10^5^	1.70 × 10^4^
*k_d_*_1_ (s^−1^)	7.12 × 10^−2^	5.57 × 10^−1^
*k_a_*_2_ (s^−1^)	1.42 × 10^−2^	6.77 × 10^−3^
*k_d_*_2_ (s^−1^)	7.99 × 10^−4^	1.64 × 10^−2^
*K_d_* (m)	1.43 × 10^−8^	2.31 × 10^−5^
*R*_max_ (RU)	15.8	46.7
χ^2^ (RU^2^)	1.43 × 10^−1^	3.35 × 10^−1^

## Discussion

Phosphatases are well-known as challenging targets for small molecules; however, substantial progress has been made in improving the tractability of this enzyme class (5, 6, 52, 53). Several screening assays have been developed to identify WIP1 inhibitors (29, 45, 46, 54, 55) and enabled the discovery of CCT007093 (46), SPI-001 (56) and its analogue SL-176 (57), and GSK2830371 (29). The inhibitor SPI-001 is 50-fold more selective toward WIP1 than PPM1A, and it has been reported that both SPI-001 and SL-176 inhibit proliferation of a cell line overexpressing WIP1 (56, 57). However, further studies are needed to evaluate the selectivities and *in vivo* activities of these compounds (17). The selectivity of GSK2830371 has been extensively investigated, with no activity observed against a panel of 21 phosphatases. Also, chemoproteomic studies demonstrated that GSK2830371 binds directly to WIP1 (29), which is supported by our SPR studies. Although inhibition of WIP1 by GSK2830371 is highly potent *in vitro* (IC_50_ = 6 nm) with good efficacy in WT P53 tumor cell lines, the compound exhibits poor pharmacokinetics with a short half-life *in vivo* (29). Unfortunately, attempts to overcome this problem through modification of the scaffold have resulted in reduced potency, with the chirality of the central l-cyclohexyl alanine moiety being crucial for the inhibitory activity (29, 30). Structure-activity relationship studies based on an analogue of GSK2830371 were performed at three positions of the scaffold. All modifications to the central position resulted in a complete loss of WIP1 inhibition, while a few modifications to the other positions, although tolerated, did not significantly improve the activity (30). Therefore, robust assays suitable for HTS are needed for the discovery of selective WIP1 inhibitors with improved pharmacokinetics. Additionally, the identification of small-molecule activators of the WIP1 phosphatase, which have not yet been reported, will enable proof-of-concept studies as a potential therapeutic strategy for P53-negative tumors. Indeed, activation of WIP1 in the absence of P53 has been shown to sensitize chemotherapy-resistant cancer cells to treatment, while protecting normal tissues from the therapy's side effects (24–26).

This article describes the development, optimization, and validation of physiologically relevant orthogonal WIP1 activity assays that are suitable for HTS. RapidFire MS is a sensitive label-free detection technology that is well-suited for *in vitro* enzymatic assays. As such, RapidFire MS assays have been developed to identify small-molecule modulators for a range of enzymes, including LRKK2 (58), sphingosine kinase (59), arginase II (60), prostaglandin E synthase (61), kynurenine aminotransferase II (62), kynurenine 3-monooxygenase (63), histone lysine demethylase (64), and HIV-1 protease (65). To the best of our knowledge, this study describes the first application of RapidFire MS to phosphatases in general and WIP1 in particular. Under conditions in which less than 20% of the substrate is consumed, the optimized assay enables a sensitive measurement of the dephosphorylated P53 peptide reaction product with an 80-fold signal-to-background ratio. The assay was validated by a pilot-scale screen of the NPC library using qHTS with five concentrations and showed excellent performance statistics, with an average Z′-factor value of 0.73 and an average CV of 8.55% for the vehicle control among forty 384-well plates. The hit rate was 3.2% with a threshold of 50% inhibition. The application of orthogonal activity assays to HTS both increases the evidence for genuine activity against the target and minimizes the probability of selecting false-positive hits, because the complementary assays are susceptible to different types of interferences (61, 62, 65). For an orthogonal readout, a red-shifted fluorescence assay was developed using the rhodamine-labeled phosphate binding protein described previously (47, 48). A similar reagent with a green fluorescence emission (λ_Ex_ = 430, λ_Em_ = 450) was also developed by the Webb laboratory (66) and is commercially available under the name Phosphate Sensor from Life Technologies. For HTS, red-shifted assays are preferable due to the substantially lower fluorescence interference by library compounds (67). The optimized Rh-PBP assay provides a sensitive and real-time readout of WIP1 enzymatic activity within the early linear phase of the reaction. Validation of the assay by qHTS of the NPC library at three doses demonstrated excellent performance statistics, with an average Z′-factor value of 0.84 and a CV of 4.77% for the vehicle control among six 1,536-well plates. While providing a sensitive real-time readout of WIP1 enzymatic activity, this orthogonal detection technology measures the WIP1 reaction product, P_i_. Thus, the RapidFire MS and Rh-PBP WIP1 assays are well-positioned to provide complementary evidence for small-molecule modulators of WIP1, while helping to identify compounds that interfere with the individual assay technologies. At 4.0%, the hit rate for the Rh-PBP assay was slightly higher than that of the RapidFire MS assay, which might be anticipated due to the fluorescence readout. Fluorescence interference is reduced by the use of a red-shifted emission signal as well as the rate determination by real-time activity measurements. Also, assay plates are measured following compound addition, but prior to reaction initiation with the substrate, to identify compounds which might interfere with the florescence measurement. For the NPC screen, this pre-read flagged 72 compounds (2.8%) that substantially altered the baseline fluorescence. Fifty-eight cherry picks were common to both RapidFire MS and Rh-PBP primary screens, of which only 34 were confirmed by both assays when re-tested at 11 doses (a final 1.3% hit rate). The activities of most compounds (NCGC00185994 is an exception, see below) were also confirmed by the Rh-PBP assay when the P53 substrate was replaced with the P38 substrate. Compounds with intractable mechanisms of inhibition, such as chemical reactivity and compound aggregation, are undesirable yet would be expected to show activity with both assay formats. Such compounds can be flagged in secondary assays and follow-up studies. The Amplex Red counter screen (51) successfully identified 10 redox active compounds among the cherry picks.

Among the confirmed cherry picks without redox activity, five compounds were selected for further characterization: a C10–16-alkylbenzenesulfonic acid (NCGC00244580), GW7647 (NCGC00015480), MK-886 (NCGC00162249), troglitazone (NCGC00161599), and 10058-F4 (NCGC00185994). These compounds were tested by an additional orthogonal BIOMOL Green WIP1 activity assay with a colorimetric readout, and an orthogonal phosphopeptide substrate derived from ATM. Interestingly, all compounds fully inhibited WIP1 by the BIOMOL Green assay except for 10058-F4, which only showed partial activity at the highest concentrations. Notably, 10058-F4 was completely inactive when the P53 substrate was replaced with the P38 substrate for the Rh-PBP assay. Together, these results suggest that the activity of 10058-F4 might be substrate-dependent. Studies are currently in progress to further evaluate this potentially interesting finding.

While each molecule binds to WIP1, the SPR data indicate that inhibitors NCGC00244580, NCGC00015480, NCGC00162249, and NCGC00161599 are likely to form aggregates with the protein and may inhibit *via* nonspecific interactions. As each of these inhibitors possess hydrophobic entities, it is not surprising that they could be promoting nonspecific aggregation. In contrast to the first four inhibitors, 10058-F4 (5-[(4-Ethylphenyl)methylene]-2-thioxo-4-thiazolidinone) does bind to WIP1 stoichiometrically with a *K_d_* of 23 μm. This molecule has been reported to specifically inhibit c-Myc (68), affect multiple cellular activities in acute myeloid leukemia (AML) (69), as well as enhance chemosensitivity and induce apoptosis of leukemic cells (70, 71). It is important to note, however, that 10058-F4 is a 5-arylmethylidenerhodanine, a class of molecules that tends to show activity in many high-throughput screens and which is often classified with pan assay interference compounds (PAINS) (72). It is possible that nucleophiles (such as the sulfur in cysteine) react with the electrophilic exocyclic double bond in 10058-F4 *via* a Michael addition, and there is evidence that such additions may be reversible. In a study of HCV RNA polymerase inhibitors, an analog of a 5-arylmethylidenerhodanine was observed as a covalent adduct to a cysteine sidechain in the crystal structure, yet the inhibition was reversible in solution (73). It is possible that 10058-F4 inhibits WIP1 by a similar type of mechanism.

The compound NCGC00244580 is a C10–16-alkyl derivate of benzenesulfonic acid. Phosphate groups that are targeted by phosphatases can be subjected to bioisosteric replacements to develop molecules which can interfere with kinase- or phosphatase-related cell signaling events (74), and it has been shown that sulfur-containing (SO_3_H) phosphate bioisosteres can be successfully employed in the development of protein tyrosine phosphatase inhibitors (74, 75). In addition, our team has shown that based on a P38 substrate peptide, cyclic thioether peptide inhibitors can be designed with a sequence cyclo(M-pS-I-pY-VAC) and cyclo(F-pS-I-pY-DDC), which could effectively inhibit WIP1 activity with a *K_i_* of 5 and 0.1 μm respectively (27, 28). Indeed, it has been observed that, while pT-X-pY is a physiologic substrate of WIP1, changing the pT to pS while keeping pY intact in a peptide sequence negatively affects WIP1 activity. Moreover, it was also demonstrated that pY contributes to the inhibitory activity against WIP1 (28). Therefore, it is possible that the benzenesulfonic acid acts as a pY mimic of the cyclic peptide to inhibit WIP1 activity; however, our results also support inhibition by nonspecific interactions.

The purpose of this study was to validate orthogonal WIP1 activity assays that utilize physiologically relevant phosphopeptide substrates to enable the discovery of small-molecule modulators by HTS. WIP1 is a potential therapeutic target and these assays can facilitate the discovery of new modulator scaffolds that might be amenable to optimization for better pharmacokinetic properties. Pilot-scale qHTS of the NPC library demonstrated the excellent performance of the complementary assays and similarities among hits. Among the confirmed cherry picks there was close agreement between the activities measured in 11-point dose response. The implementation of orthogonal readouts reduced the prevalence of interference compounds, while counter-screens and follow-up studies enable identification of compounds with intractable mechanisms of action, such as nonspecific reactivity, redox or aggregation. These studies provide a basis for the discovery of selective WIP1 modulators, both inhibitors and activators, by establishing a testing funnel of assays to identify and triage hits from small-molecule screens that can also be easily adapted as a discovery strategy for modulators of other phosphatases.

## Experimental procedures

### Chemicals, reagents and libraries

The components of the WIP1 reaction buffer were obtained from various sources, including 1 m TRIS pH 7.5 (Invitrogen, Cat# 15567–027), 1 m MgCl_2_ (Quality Biological, Cat# 351–033-721), Tris(2-carboxyethyl)phosphine hydrochloride (TCEP, Sigma, Cat# C4706), Ethylene glycol-bis(2-aminoethylether)-N,N,N′,N′-tetraacetic acid (EGTA, Fluka, Cat# 03778), and BSA (Sigma Aldrich, Cat#A7906–100G). Dimethyl sulfoxide (DMSO) was purchased from Sigma (Cat# D8418). The test compounds screened were from the NCATS Pharmaceutical Collection of 2,816 compounds (49). The collection includes replicates of some compounds as internal controls and a total number of 2,535 unique compounds. The control compound GSK2830371 was obtained from Sigma (Cat# SML-1048).

### Peptide synthesis and purification

The peptides used in this study (**1–6**) were generated manually by the standard solid phase synthesis method utilizing 9-fluorenylmethoxycarbonyl (Fmoc)/tert-butyl or tert-butoxycarbonyl (Boc)/benzyl chemistry. The amino acid sequence corresponding to each peptide was assembled on Wang resin with *N*^α^-Fmoc-protected amino acid derivatives whereas phosphorylated Ser, Thr, and Tyr were incorporated as Fmoc-Ser[PO(OBzl)OH]-OH, Fmoc-Thr[PO(OBzl)OH]-OH, and Fmoc-Tyr[PO(OBzl)OH]-OH, respectively. The coupling reactions were conducted by means of the HOBt/HBTU, DIEA in NMP method. Each coupling reaction was performed for 1 h and the Fmoc group was eliminated by treating the resin with 20% piperidine in NMP for 10 min. Peptide **4** (VEPPL-S-QETF*S) was synthesized with ring ^13^C_6_-labeled l-phenylalanine, where F* designates the ring-^13^C_6_-labeled phenylalanine, 99%, (Cambridge Isotope Laboratories, Inc.). All peptides were synthesized with free N- and C termini, including the P38 peptides: di-phosphorylated peptide **1** (TDDEM-pT-G-pY-VAT) and mono-phosphorylated peptide **2** (TDDEM-pT-GYVAT), the P53 peptides: phosphorylated peptide **3** (VEPPL-pS-QETFS), heavy labeled nonphosphorylated peptide **4** (VEPPL-S-QETF*S) and nonphosphorylated peptide **5** (VEPPL-S-QETFS), and the ATM phosphorylated peptide **6** (AFEEG-pS-QSTTIGY).

Cleavage of the peptide from the resin and concomitant elimination of the side chain-protecting groups was accomplished with a TFA/water/triisopropylsilane mixture (92.5/5/2.5, v/v) or a trifluoromethanesulfonic acid/TFA/water/triisopropylsilane mixture (10/82.5/5/2.5, v/v) for 2 h at room temperature. After the resin had been removed by filtration, the filtrate was concentrated by flushing with nitrogen gas and crude peptides were precipitated with diethyl ether. Crude peptides were purified using reversed-phase HPLC (RP-HPLC) on a preparative C4 column (BioAdvantage Pro 300, Thomson Liquid Chromatography) with a water/acetonitrile solvent system containing TFA. Purified peptides were characterized by MALDI-TOF MS (MALDI microMX, Waters) and RP-HPLC on an analytical C18 column (Eclipse XDB-C18, Agilent). The purity of all peptides was found to be >98%.

### Expression and purification of WIP1(1–420)

The WIP1(1–420) coding sequence was subcloned into the pE-SUMOstar vector (LifeSensors, Cat# 1106) and co-transformed into *Escherichia coli* BL21(DE3) with pGro7(groES-groEL) (Takara, Cat# 3340). The cells were grown in LB media containing 0.5 mg/ml L(+)arabinose, 10 μg/ml chloramphenicol, and 100 μg/ml ampicillin at 37 °C until the OD_600_ reached 0.5–0.6. Protein expression was induced by the addition of 0.3 mm IPTG at 30 °C overnight. Cells were harvested by centrifugation and then resuspended in PBS pH 7.5, containing 2 mm β-mercaptoethanol, 2 mm MgCl_2_, 20% glycerol, 0.2% Triton X-100, 0.5% (w/v) CHAPS supplemented with cOmplete EDTA-free protease inhibitor (Roche, Cat# 05056489001). The cells were lysed by French press and the lysate was cleared by centrifugation (40,000 × *g*, 40 min, 4 °C). The protein was purified using TALON metal affinity resin (Takara, Cat# 635502). The pooled elution fractions from the TALON metal affinity column were dialyzed against buffer A (20 mm Tris-HCl, pH 7.5, 2 mm MgCl_2_, 0.02% β-mercaptoethanol, 20% glycerol, and 0.1 mm EGTA) supplemented with 150 mm NaCl. Following overnight dialysis, the sample was incubated with SUMOstar protease 1 (LifeSensors, Cat# 4110) for 2 h at 30 °C and then loaded onto a SP-Sepharose column that was pre-equilibrated with buffer A. The column was subsequently washed with the same buffer, and the protein was eluted by using a 150–400 mm NaCl gradient. The pooled fractions from SP-Sepharose were dialyzed against buffer A supplemented with 300 mm NaCl. The protein was >95% pure as determined by SDS-PAGE followed by Coomassie Blue staining. Protein concentration was determined by optical spectrometry (NanoDrop, Thermo Scientific) using ϵ_280_ = 50,420 m^−1^ cm^−1^.

### Expression and purification of PBP (A17C, A197C)

The pET22b plasmid encoding the protein was transformed into *Escherichia coli* BL21(DE3), and the cells were grown in LB media containing 100 μg/ml ampicillin at 37 °C until the OD_600_ reached a value of 1.0. Protein expression was induced by the addition of 0.4 mm IPTG at 37 °C for 3 h. Cells were harvested by centrifugation. The harvested cell pellets were resuspended in 20 mm Tris-HCl, pH 8.0, containing 1 mm EDTA, 5 mm DTT, and cOmplete EDTA-free protease inhibitor (Roche, Cat# 05056489001). The cells were lysed by French press and the lysate was cleared by centrifugation (100,000 × *g*, 1 h, 4 °C). Ammonium sulfate was added to the supernatant up to 50% saturation with stirring at 4 °C. After 2 h, insoluble materials were removed by centrifugation at 40,000 × *g* for 30 min at 4 °C. Additional ammonium sulfate was added to the supernatant up to 90% saturation with stirring at 4 °C and centrifugation was repeated after overnight stirring. The pellet was resuspended in buffer: 20 mm Tris-HCl, pH 8.0, containing 1 mm EDTA, and 1 mm DTT, and extensively dialyzed against the same buffer. The protein was then loaded onto a Q-Sepharose column that was pre-equilibrated with buffer: 10 mm Tris-HCl, pH 8.0, containing 1 mm DTT. The column was subsequently washed with the same buffer, and the protein was eluted by using a 0–1 M NaCl linear gradient. The pooled fractions from Q-Sepharose were concentrated and gel filtration was performed using Superdex-75 16/60 (GE-Healthcare) under FPLC conditions in the presence of buffer: 10 mm Tris-HCl, pH 8.0 containing 200 mm NaCl, 5 mm DTT, and 10% glycerol. The protein was >95% pure as determined by SDS-PAGE followed by Coomassie Blue staining. Protein concentrations were determined by optical spectrometry (NanoDrop, Thermo Scientific) using ϵ_280_ = 60,880 m^−1^ cm^−1^.

### Phosphate Binding Protein rhodamine conjugate synthesis

6-chloroacetamidotetramethylrhodamine was synthesized from 6-aminotetramethyl rhodamine (Setareh Biotech, Eugene, OR) according to the previously published procedure (76). 6-Iodoacetamidotetramethylrhodamine (6-IATR) was prepared from the precursor 6-chloroacetamidotetramethylrhodamine using a modified method. The reaction was heated at 55 °C for 15 h in the presence of sodium iodide and ethanol with 70% conversion yield. The crude 6-IATR was purified using a RediSep Rf C-18 column with a 100% water wash (7 column volumes (CV), 25 min) followed by a gradient to 100% acetonitrile (17 CVs, 60 min). The solvents were removed under reduced pressure and trace amounts of water were co-evaporated three times with acetonitrile. The product was analyzed by LC MS (Agilent 1290 Series 6130 Quadrupole) using a Poroshell 120 SB-C18 column (4.6 × 50 mm) with a flow rate of 1.22 ml/min and with a 5% B isocratic hold for 0.3 min (A: 0.05% TFA in H_2_O; and B: 0.05% TFA in CH_3_CN) followed by a gradient from 5% B to 95% B over 3.5 min. The retention time of 6-IATR is 3.24 min. *m/z* (APCI): [M+H]^+^ 570.2.

Labeling of the Phosphate Binding Protein A17C, A197C mutant (PBP) with 6-IATR was performed following the protocol established by Webb (47) with a few modifications. PBP was reduced with a 10-fold excess of dl-DTT and the reduced PBP was purified through a PD-10 desalting column (GE Healthcare) equilibrated with 10 mm Tris-HCl, 1 mm EDTA, pH 8. The PBP aliquot (100 μm) was then incubated with 200 μm 7-methylguanosine and 0.2 units/ml bacterial nucleoside phosphorylase (Sigma Aldrich, St Louis, MO) to remove contaminating P_i_. 6-IATR was dissolved in DMF and added at a 4-fold excess over PBP cysteines. The reaction was protected from light and mixed at room temperature for 2 h before quenching the unreacted dye with an excess of sodium 2-mercaptoethanesulfonate. The mixture was filtered through a 0.22 μm syringe filter and Amicon Ultra 10K MWCO centrifugal filters (Millipore) were used to remove free dye and concentrate the protein. Doubly labeled Rh-PBP was purified using a HiPrep Q FF 16/10 column (GE Healthcare) at 2 ml/min with a 40 CV gradient from 0 to 200 mm NaCl in 10 mm Tris pH 8. The purified Rh-PBP was then concentrated with a 10K MWCO centrifugal filter. Labeling was confirmed by MS (Waters Xevo G2-XS QTof). Deconvoluted mass: unmodified PBP 34,616.2 Da and doubly-labeled 35,499.3 Da.

### Rhodamine-labeled Phosphate Binding Protein (Rh-PBP) WIP1 activity assay

The Rh-PBP WIP1 activity assays were performed in black, medium binding, solid bottom 1,536-well plates (Greiner, Cat# 789176-F). First, 13 nl of library compounds, GSK2830371 or DMSO control were pin-transferred (Kalypsis) into 3 μl of reaction buffer (with the P53 substrate peptide **3**: 50 mm Tris-HCl pH 7.5, 30 mm MgCl_2_, 1 mm TCEP, 0.1 mm EGTA, 75 μg/ml BSA; with the P38 substrate peptide **1**: 50 mm Tris-HCl pH 7.5, 30 mm MgCl_2_, 1 mm TCEP, 0.1 mm EGTA, 0.01% Tween 20) containing WIP1 (with the P53 substrate: 6.7 nm [5 nm final]; with the P38 substrate: 7.2 nm [5.4 nm final]). A phosphate standard curve [0, 2, 3, 5, 7, 8, 9, 10 μm; *n* = 4] was included in column 1 to enable quantification of phosphate from the fluorescence intensity. Plates were then incubated for 1 h at room temperature with the control and library compounds. Prior to initiating the reaction, a detection measurement was made (λ_Ex_ = 525 nm, λ_Em_ = 598 nm) with a ViewLux uHTS microplate imager (PerkinElmer) to identify autofluorescent compounds. The exposure time was 2 s with an excitation energy of 1,000 and no image binning. The narrow band interference filter for excitation was 525 nm ± 10 nm (525/20) and the emission filter was a 598 nm ± 12.5 nm (598/25). The WIP1 reaction was initiated by the addition of 1 μl phosphopeptide substrate **1** or **3** (40 μm [10 μm final]). Immediately following substrate addition, fluorescence was measured at 1 min intervals for 11 min.

### RapidFire Mass Spectrometry assay

RapidFire MS screening was performed in 384-well round bottom polypropylene plates (Thermo Scientific, Cat# 264573). Assay controls included both the DMSO vehicle (high signal control) and 10 μm GSK2830371 (100% inhibition, low signal control). The enzyme and substrate (peptide 3) solutions were prepared as 1.25x and 5x solutions, respectively, in assay buffer: 50 mm Tris-HCl pH 7.5, 30 mm MgCl_2_, 1 mm TCEP, 0.1 mm EGTA, 75 μg/ml BSA. Heavy-labeled product peptide **4** was prepared in 0.1% formic acid. Final concentrations of each reagent were as follows: 10 nm WIP1, 2 μm substrate peptide **3**, 1 μm heavy-labeled product peptide **4**. All steps were performed at room temperature. All solutions were dispensed with a BioRaptr work station (Beckman Coulter). 8 μl of WIP1 solution were added in 384-well assay plates, then 63 nl of controls and compounds were added in the plate with a pintool work station (Kalypsis). Plates were then incubated at room temperature for 1 h, after which the reaction was initiated by adding 2 μl of substrate peptide **3** solution. 5 min after substrate addition, the reaction was quenched with 2 μl of 0.5% formic acid in H_2_0. 38 μl of heavy-labeled product peptide **4** were then dispensed, for a final volume of 50 μl in each well. Finally, plates were sealed with Agilent PlateLoc Thermal Microplate Sealer and stored at 4 °C until the RapidFire MS run. The RapidFire system, which conducts high speed solid phase extraction (sampling, loading, washing, and injection) through multiple pumps and valve system, delivers eluted analytes directly to the mass spectrometer (RapidFire MS) (77). Here, an Agilent RapidFire 360 automated extraction system with three HPLC pumps coupled to an Agilent 6470 triple quadrupole mass spectrometer (Agilent, Santa Clara, CA) equipped with a Agilent Jet Stream Electrospray Ionization (AJS ESI) interface source was used for the compound screen assay. Agilent RapidFire 4.0 and Agilent MassHunter B.08.00 programs were used for instrument control and data acquisition, respectively. The RapidFire MS system was equipped with a C4 Type A solid-phase extraction (SPE) cartridge. There are two major solvents for the RapidFire MS, solvent A (water with 0.1% (v/v) formic acid) was used for sample loading and washing; solvent B (80% Acetonitrile, 20% water, 0.1% (v/v) formic acid) was used for sample elution. In brief, samples were first aspirated onto the sample collection loop (10 μl) under vacuum directly from multiwell plates. A fixed time of 600 ms was defined as the maximum aspiration time with a liquid sensor for detecting whether the loop is full (state 1). The 10 μl sample was loaded onto the C4 cartridge and washed by pump 1, using solvent A at a flow rate of 1.5 ml/min for 3,000 ms (state 2). The retained analytes on the C4 cartridge were then directly eluted to the mass spectrometer by pump 3, using solvent B at a flow rate of 1.25 ml/min for 3,500 ms (state 4). After elution, the system was re-equilibrated by pump 1, using solvent A at a flow rate of 1.5 ml/min for 2,500 ms (state 5). State 3 was designed for an extra wash, which was not required in the optimized method. The sample collection loop was washed with solvent B at a flow rate of 1.25 ml/min for 3,500 ms using pump 2, while the sample was under elution (state 4). The entire sampling cycle was ∼10 s per well. The Agilent 6470 QQQ mass spectrometer was equipped with an AJS ESI. Before use, the instrument was tuned and calibrated in positive mode. To achieve the best selectivity and sensitivity, the mass spectrometer was operated in multiple reaction monitoring (MRM) mode with Q1 resolution set to unit and Q3 resolution set to wide, delta EMV at 200 V. The gas temperature, gas flow, sheath gas temperature, and sheath gas flow were set to 325 °C, 10 L/min, 400 °C, and 11 L/min, respectively. Electrical voltages were optimized for the capillary voltage at +3,500 V, nebulizer voltage at +1,000 V. LOQ was determined from triplicate experiments each with 4 technical replicates at 0, 24, 48 and 72 h after quenching and analyzed together (*n* = 12) using the calculation method recommended by Shrivastava *et al.* (78), dividing 10 S.D. of the lowest concentration by the slope of the regression.

### RapidFire Mass Spectrometry data processing

The acquired data from RapidFire MS was automatically split into plate well location corresponding data format and imported into Agilent MassHunter Quantitative Analysis (B.07.00) for further MRM extraction and integration analysis. Peptide **4** (same peptide sequence as peptide **5**, but ^13^C_6_-labeled) was spiked into each well at a final concentration of 1 μm, which was used as the internal standard for normalizing the MS signals across different batches. Integrated area ratios of peptide **5** divided by peptide **4** were used for further informatics analysis. The NPC library was screened in 5-pt dose response ranging from 63 μm to 0.1 μm with 1:5 dilution. To determine compound activities from the qHTS RapidFire MS assay, the concentration-response data for each sample was plotted and modeled by a four-parameter logistic fit, yielding EC_50_ and efficacy (maximal response) values. Raw plate reads for each titration point were first normalized relative to the positive control (10 μm GSK2830371, -100% activity, full inhibition) and DMSO only wells (basal, 0% activity).

### Steady-state kinetics and WIP1 in vitro inhibition assay

Initial rates of WIP1 phosphatase activity were determined by incubating WIP1(1–420) (26 nm) with various concentrations of [h-ATM (1976–1986-GY, 1981pS)] peptide **6** in the buffer: 50 mm Tris-HCl (pH = 7.5), 30 mm MgCl_2_, 0.1 mm EGTA, 0.1 mg/ml BSA, 1 mm CHAPS and 1 mm DTT at 30 °C for 7 min. The phosphate reaction product was measured using the BIOMOL green assay (Enzo Lifesciences), following the manufacturer's protocol. To estimate *K_m_* and *k_cat_* values, the initial rates were fit to the Michaelis-Menten equation in GraphPad Prism 7.01. Inhibition of phosphatase activity of WIP1(1–420) (26.0 nm) were performed as described previously (30). Inhibition of phosphatase activity was determined in the above buffer and 1% DMSO using 90.0 μm peptide **6**.

### Amplex Red (10-Acetyl-3,7-dihydroxyphenoxazine) assay

The assay was adapted from a previously described protocol to assess redox cycling of compounds in the presence of reducing agents (51). 13 nl of compounds (or DMSO control) were pin-transferred into 2.5 μl HBSS (Thermo Fisher; containing 1.26 mm CaCl_2_, 0.49 mm MgCl_2_, 1 g/l d-glucose) in black 1,536 well plates. Compound fluorescence was measured immediately (READ 0) using a ViewLux uHTS microplate imager (PerkinElmer) equipped with Ex: 525/20 and Em: 598/25 filters. 2.5 μl of a 2X Amplex Red solution [100 μm Amplex Red (Cayman Chemical, Ann Arbor), 200 μm DTT (Thermo Fisher) and 2 U/ml horse radish peroxidase (Sigma-Aldrich); diluted in HBSS and protected from light] were added to each well. Fluorescence was measured after a 15 min incubation at room temperature (READ 1), using ViewLux settings identical to READ 0. Activity was calculated using corrected fluorescence values (READ 1 minus READ 0), which were compared with control samples (negative = vehicle; positive = 46 μm walrycin B).

### Surface plasmon resonance binding measurements

SPR measurements were conducted on a Biacore 8K instrument (GE Healthcare). WIP1 was immobilized on a CM5 sensorchip at 25 °C using standard amine coupling chemistry at a flow rate of 5 μl/min. Briefly, the dextran surface was activated by a 10-min injection of a 1:1 ratio of 0.4 m EDC (1-ethyl-3-(3-dimethylaminopropyl)carbodiimide hydrochloride) and 0.1 m NHS (N-hydroxysuccinimide) followed by a 7-min injection of WIP1 in 20 mm Bis-Tris pH 6.5 at a concentration of 30 μg/ml. All unreacted groups on the surface were blocked with a 7-min injection of 1 m ethanolamine at pH 8.5. HBS-*p* + (10 mm HEPES, 150 mm NaCl, 0.05% (v/v) surfactant P20, pH 7.4) with 10% glycerol was used as the running buffer for immobilization. A Zeba^TM^ Micro Spin Desalting Column 7K MWCO (Thermo Scientific) was used to buffer exchange single-use aliquots of protein from the storage buffer (20 mm Tris-HCl, pH 7.5, 0.1 mm EGTA, 0.02% β-mercaptoethanol, 300 mm NaCl, 2 mm MgCl_2_ in 20% glycerol) into the running buffer. Buffer-exchanged protein was then diluted into the 20 mm Bis-Tris pH 6.5 buffer immediately before immobilization. An immobilization level between ∼3,000–7,000 RU was achieved. Binding measurements were conducted at 25 °C in HBS-*p* + with 10% glycerol and 1% DMSO. To validate the assay GSK2830371 was used as the positive control. All binding measurements were conducted on the same day as immobilization using a flow-rate of 45 μl/min. For GSK2830371 and NCGC00185994 the data were fit with a two-state kinetic binding model. The binding behavior of the other test analytes deviated from stoichiometric binding such that a reliable fit to the data could not be obtained.

### Data analysis

Data normalization and curve fitting were performed using in-house informatics tools. Briefly, raw plate reads for each titration point were first normalized relative to the DMSO-only wells (100% activity) and 100% inhibition control wells (0% activity), and then corrected by applying a plate-wise block pattern correction algorithm to remove any plate edge effects and systematic background noise. Active compounds in the primary HTS were defined as having a maximum response ≤ 50%. To determine compound activities in the 11-point qHTS, the concentration-response data for each sample was plotted and modeled by a four parameter logistic fit yielding IC_50_ and efficacy (maximal response) values as previously described (50). The activities were designated as class 1–4 according to the type of concentration-response curve observed. Active compounds were defined as having concentration-response curves in the classes of 1–3. The promiscuity score for each compound was defined as (number of assays that the compound is active)/(total number of assays that the compound was tested in). Any compound with a promiscuity score higher than 0.2 was considered as a “frequent hitter” to be eliminated from the follow-up studies.

## Author contributions

V. C., H. D. T., S. D., S. J. M., M. R. W., M. D. H., E. A., D. H. A., and N. P. C. conceived and initiated the research; V. C., D. T., Y. F., H. D. T., S. D., Y. W., H. S., C. A. L., S. J. M., K. L., Z.-D. S., O. V., R. E., L. K. B., M. J. H., M. S., M. D. H., E. A., D. H. A., and N. P. C. conducted the research; all authors analyzed and discussed the data and contributed to and approved of the final manuscript.

## Footnotes

5 The abbreviation used is:

WIP1: WT P53-Induced Phosphatase 1.
